# Levosimendan in intensive care and emergency medicine: literature update and expert recommendations for optimal efficacy and safety

**DOI:** 10.1186/s44158-021-00030-7

**Published:** 2022-01-24

**Authors:** M. Girardis, D. Bettex, M. Bojan, C. Demponeras, S. Fruhwald, J. Gál, H. V. Groesdonk, F. Guarracino, J. L. Guerrero-Orriach, M. Heringlake, A. Herpain, L. Heunks, J. Jin, D. Kindgen-Milles, P. Mauriat, G. Michels, V. Psallida, S. Rich, S-E Ricksten, A. Rudiger, M. Siegemund, W. Toller, S. Treskatsch, Ž. Župan, P. Pollesello

**Affiliations:** 1 Anesthesiology Unit, University Hospital of Modena, University of Modena & Reggio Emilia, Modena, Italy; 2 Cardio-Surgical Intensive Care Unit, Institute of Anesthesiology, University Hospital Zurich and University of Zurich, Zurich, Switzerland; 3 Anesthesiology and Intensive Care, Hôpital Marie Lannelongue, Le Plessis-Robinson, France; 4 Intensive Care Unit, Sotiria General Hospital, Athens, Greece; 5 Department of Anaesthesiology and Intensive Care Medicine, Division of Anaesthesiology for Cardiovascular Surgery and Intensive Care Medicine, Medical University of Graz, Graz, Austria; 6 Department of Anaesthesiology and Intensive Therapy, Semmelweis University, Budapest, Hungary; 7 Clinic for Interdisciplinary Intensive Medicine and Intermediate Care, Helios Clinic, Erfurt, Germany; 8 Dipartimento di Anestesia e Rianimazione, Azienda Ospedaliero-Universitaria Pisana, Pisa, Italy; 9 Institute of Biomedical Research in Malaga, Department of Anesthesiology, Virgen de la Victoria University Hospital, Department of Pharmacology and Pediatrics, School of Medicine, University of Malaga, Malaga, Spain; 10 Department of Anesthesiology and Intensive Care Medicine, Heart and Diabetes Center, Mecklenburg-Western Pomerania, Karlsburg Hospital, Karlsburg, Germany; 11 Department of Intensive Care, Erasme University Hospital, Université Libre De Bruxelles, Brussels, Belgium; 12 Department of Intensive Care, University Medical Center, University of Amsterdam, Amsterdam, the Netherlands; 13 The Fourth Hospital of Changsha, Changsha City, Hunan Province People’s Republic of China; 14 Interdisciplinary Surgical Intensive Care Unit, Department of Anesthesiology, Medical Faculty, Heinrich Heine University, Dusseldorf, Germany; 15 Department of Anaesthesia and Critical Care, University of Bordeaux, Haut-Levêque Hospital, Pessac, France; 16 Clinic for Acute and Emergency Medicine, St. Antonius Hospital, Eschweiler, Germany; 17 Intensive Care Unit, Agioi Anargyroi Hospital, Athens, Greece; 18 Northwestern University Feinberg School of Medicine, Chicago, IL USA; 19 Department of Anesthesiology and Intensive Care Medicine, Sahlgrenska Academy, University of Gothenburg, Gothenburg, Sweden; 20 Department of Medicine, Limmattal Hospital, Limmartal, Switzerland; 21 Intensive Care Unit, Department Acute Medicine, University Hospital Basel, Basel, Switzerland; 22 Charité—Universitätsmedizin Berlin, Corporate Member of Freie Universität and Humboldt Universität zu Berlin, Department of Anesthesiology and Intensive Care Medicine, Charité Campus Benjamin Franklin, Berlin, Germany; 23 Department of Anesthesiology, Intensive Care Medicine and Pain Therapy, KBC Rijeka, Rijeka, Croatia; 24 Critical Care, Orion Pharma, P.O. Box 65, FIN-02101 Espoo, Finland

**Keywords:** Intensive care unit, Emergency medicine, Acute cardiac care, Haemodynamic, Inotrope, Inodilator, Levosimendan, Renal dysfunction

## Abstract

The inodilator levosimendan, in clinical use for over two decades, has been the subject of extensive clinical and experimental evaluation in various clinical settings beyond its principal indication in the management of acutely decompensated chronic heart failure. Critical care and emergency medicine applications for levosimendan have included postoperative settings, septic shock, and cardiogenic shock. As the experience in these areas continues to expand, an international task force of experts from 15 countries (Austria, Belgium, China, Croatia, Finland, France, Germany, Greece, Hungary, Italy, the Netherlands, Spain, Sweden, Switzerland, and the USA) reviewed and appraised the latest additions to the database of levosimendan use in critical care, considering all the clinical studies, meta-analyses, and guidelines published from September 2019 to November 2021. Overall, the authors of this opinion paper give levosimendan a “should be considered” recommendation in critical care and emergency medicine settings, with different levels of evidence in postoperative settings, septic shock, weaning from mechanical ventilation, weaning from veno-arterial extracorporeal membrane oxygenation, cardiogenic shock, and Takotsubo syndrome, in all cases when an inodilator is needed to restore acute severely reduced left or right ventricular ejection fraction and overall haemodynamic balance, and also in the presence of renal dysfunction/failure.

## Introduction

The calcium-sensitizing inodilator levosimendan has been in clinical use for two decades, during which time it has been the subject of extensive clinical and experimental evaluation in numerous clinical settings [1].

Beyond its principal indication in the management of acutely decompensated chronic heart failure (AHF), levosimendan has been evaluated for its potential in a range of emergency and critical care applications, including postoperative (predominantly cardiac surgery) situations, sepsis/septic shock, renal impairment/failure and cardiogenic shock (CS). Previous reviews and expert opinion articles have summarized the clinical experience in those areas and provided recommendations for practice [2–5].

The database of experience in these areas continues to expand and it is topical and timely to revisit the previous expert opinions and reconsider current guidance on the use of levosimendan in these applications. To that end, an international task force of experts from 15 countries (Austria, Belgium, China, Croatia, Finland, France, Germany, Greece, Hungary, Italy, the Netherlands, Spain, Sweden, Switzerland, and the USA) has reviewed and appraised recent additions to the database of levosimendan use in critical illnesses. Selection criteria used for this purpose to identify suitable reports on PubMed were as follows:

- Sources restricted to the last 2 years (from September 2019 to November 2021).
- Broad inclusions centred on clinical reports, plus meta-analyses, and guidelines.
- Inclusions restricted to studies of intravenous (i.v.) levosimendan only.
- Focus on eight therapeutic settings of intensive care unit (ICU) and emergency medicine (EM), excluding hospitalization for AHF or advanced heart failure (because described in a separate update [3]). The selected areas are (1) postoperative settings, (2) septic shock, (3) weaning from ventilator, (4) weaning from veno-arterial extracorporeal membrane oxygenation (VA-ECMO), (5) cardiogenic shock, (6) Takotsubo syndrome (TTS), (7) acute cardiac care complicated by renal dysfunction/failure, and (8) pulmonary hypertension (PH) and right ventricular dysfunction.

Summaries and analyses of the data identified in this exercise are presented in a consistent structure: each section commences with the general findings from the most recent meta-analyses and/or guidelines (where available), proceeds to individual clinical studies published in the qualifying period, and ends with an expert commentary by the authors.

## Postoperative settings

### Recent meta-analyses

The dominant meta-analysis published during the last two years is the work of Weber and colleagues, who collated data from 27 randomized controlled trials (RCTs) involving a total of 3198 patients to examine the effect of levosimendan in cardiac surgery patients [6]. Pooled data from these studies, which included the placebo-controlled LEVO-CTS, CHEETAH, and LICORN trials, were used to investigate treatment effects on mortality and other outcomes.

Strong signals of treatment benefit were identified on several major outcomes, including mortality (assessed from in-hospital or 30-day data from 15 studies). Specifically, levosimendan led to a significant reduction in mortality (odds ratio [OR] 0.67; 95% confidence interval [CnI] 0.49–0.91; *p* = 0.0087) and significantly lowered the incidence of low cardiac output syndrome (LCOS) (OR 0.56; 95% CnI 0.42–0.75; *p* < 0.0001), acute kidney injury (AKI; OR 0.63; 95% CnI 0.46–0.86; *p* = 0.0039) and renal replacement therapy (OR 0.70; 95% CnI 0.50–0.98; *p* = 0.0332). Subgroup analyses indicated that patients who received levosimendan before the operation and who had a baseline left ventricular ejection fraction (LVEF) < 35% had statistically robust benefits across many of these endpoints (Table 1).

**Table 1:** Clinical outcomes among RCTs comparing prophylactic levosimendan therapy prior to cardiac surgery in high-risk patients with an ejection fraction of < 35%. In hospital/30 days follow-up. Data from Weber et al. [6]. *CI*, confidence interval; *LH*, length of hospital stay; *OR*, odds ratio, with values < 1 favouring levosimendan; *WMD*, weighted mean difference, with negative values favouring levosimendan

**Dichotomous**	**Sample size** ***(n)***	**Prevalence %** ***(n)***	**Levosimendan %** ***(n)***	**Control %** (***n***)	**OR (95% CnI)**	***X***^**2**^**-test** ***p-value***
**Mortality**	1,224	5.5% (67)	3.7% (23)	7.2% (44)	0.49 (0.29-0.83)	0.0098
**Myocardial infarction**	1,141	12.4% (141)	12.0% (69)	12.7% (72)	0.60 (0.15-2.41)	0.7795
**Low cardiac output**	1,191	19.8% (236)	15.3% (92)	24.4% (144)	0.56 (0.42-0.75)	0.0001
**Acute kidney injury**	302	11.9% (36)	7.9% (12)	16.0% (24)	0.44 (0.21-0.93)	0.0460
**Renal replacement**	1,224	4.7% (57)	3.4% (21)	5.9% (36)	0.54 (0.31-0.95)	0.0497
**Atrial fibrillation**	1,191	32.0% (381)	31.2% (187)	32.8% (194)	0.52 (0.19-1.40)	0.5812
**Prolonged inotropic support**	849	58.8% (499)	54.9% (235)	62.7% (264)	0.72 (0.55-0.95)	0.0056
**Continuous**	**Sample size (*n*)**	**WMD**		**95% CnI**		**Overall effect** ***p*–value**
**ICU stay (days)**	72	-2.21		-6.18 to 1.75		0.27
**LH (days)**	346	-3.97		-4.69 to -3.25		< 0.0001

Separately, Terbeck and colleagues, arguing from the premise that patients with heart failure may be especially susceptible to the vasodilatory effects of drugs, explored whether the use of levosimendan increases the risk of vasoplegic syndrome (i.e., severe refractory hypotension and low systemic vascular resistance in the absence of a low cardiac output [CO] state) in patients undergoing cardiac surgery with attendant cardiopulmonary bypass (CPB) [7]. All but one of the 16 included studies (14 RCTs plus two other published studies) examined the use of preoperatively administered levosimendan. Thirteen studies compared levosimendan with placebo, two with an intra-aortic balloon pump (IABP), and one with nitroglycerine. The conclusion from this work was that there is “no clinical evidence that levosimendan produces vasopressor-resistant vasoplegic syndrome”. Levosimendan increased cardiac index (CI) in almost all of the studies reviewed but did not lower systemic vascular resistance once data had been corrected for baseline differences between comparator groups.

The safety of levosimendan in the cardiac surgery setting has been examined in two recent meta-analyses [8, 9]. Notwithstanding the acknowledged limitations of both exercises, the broad conclusions of these appraisals were reassuring. Levosimendan was not associated with increased risks of re-operation for bleeding or postoperative blood loss or with increased postoperative transfusion requirements.

### Recent clinical trials

#### Levosimendan in heart transplantation studies

Immohr and colleagues [10] retrospectively examined data from 150 patients who underwent heart transplantation between 2010 and 2020 and identified 41 patients who were treated postoperatively with levosimendan, 36 of whom had postoperative primary graft dysfunction. Stratification of patients according to whether levosimendan was commenced early (≤ 48 h post-transplant [*n* = 23]) or late (> 48 h post-transplant [*n* = 18]) revealed that early commencement was associated with a range of statistically significant outcome advantages, which included decreased duration of VA-ECMO support (5.1 ± 3.5 vs 12.6 ± 9.3 days; *p* < 0.01) and decreased mortality (0.0% vs 33.3%; *p* < 0.01). Due to their shorter time on VA-ECMO, patients whose levosimendan treatment started early required fewer blood transfusions (*p* < 0.05) and had shorter ventilation times (279 ± 235 vs 428 ± 293 h; *p* = 0.03) than those whose treatment was started later. Early introduction of levosimendan was also associated with trends of reduced incidence of postoperative renal failure (69.6% vs 94.4%; *p* = 0.06) and improved survival (*p* = 0.09).

#### Coronary artery bypass grafting studies

In a pre-specified additional analysis of levosimendan in patients with left ventricular systolic dysfunction undergoing cardiac surgery requiring cardiopulmonary bypass (LEVO-CTS) trial, van Diepen et al. [11] compared treatment-related outcomes according to cardiac surgical procedure (isolated coronary artery bypass grafting [CABG] [*n* = 563], isolated valve [*n* = 97] or CABG/valve surgery [*n* = 188]).

No surgery-specific significant differences were apparent for the composite primary outcomes but, among patients undergoing isolated CABG, 90-day mortality was lower in the levosimendan group than the placebo group (2.1% vs 7.9%; hazard ratio [HR] 0.26; 95% CnI 0.11–0.64); the incidence of LCOS was also lower in the isolated CABG subset, but not significantly different in valve surgery (8.3% vs 2.0%; HR 4.10; 95% CnI 0.46–36.72) or combined procedures (10.4% vs 7.6%; HR 1.39; 95% CnI 0.53–3.64; *p* = 0.011 for interaction). LCOS incidence (12.0% vs 22.1%; OR 0.48; 95% CnI 0.30–0.76) was also significantly lower in levosimendan-treated patients undergoing isolated CABG.

Primary results from a randomized clinical trial conducted in 279 consecutive patients with LVEF < 35% undergoing CABG surgery identified no significant difference in mortality rate between patients who received 0.1 μg/kg/min levosimendan for 24 h and those assigned to an IABP, although the length of ICU stay was shorter in the levosimendan group (4.4 vs 5.2 days; *p* = 0.05) [12]. The use of levosimendan in high-risk cardiac patients was thus comparable to that of an IABP, with implications for cost of care.

In a small prospective observational study by Khaled et al. [13], 60 patients with preoperative LVEF < 35% undergoing cardiac surgery for valvular, coronary bypass, or aortic aneurysm repair were assigned to either conventional inotropes and vasoactive therapies (*n* = 30) or to levosimendan, initially as a loading dose of 6–12 μg/kg (dose depending on mean arterial pressure) over 0.5 h and then an infusion at rates of 0.05–0.2 μg/kg/min for 24 h. Improvements in LVEF (*p* = 0.002 vs control group) and other haemodynamic alterations consistent with the established profile of levosimendan were noted but there was no difference in mortality between the groups (nine deaths versus 10).

In a separate small study that involved comparison of 13 patients with LVEF < 40% prospectively treated with a 24-h levosimendan infusion (0.05–0.2 μg/kg/min, no loading dose beginning 48 h before surgery) undergoing elective CABG with 41 retrospectively identified controls, levosimendan use was associated with a significantly lower incidence of postoperative LCOS (15% vs 61%; *p* < 0.01) and a significantly shorter ICU stay (2 versus 4 days, *p* = 0.03). There was a cost-saving of ≈ €2000/patient with levosimendan that was robust in sensitivity analyses [14].

Stefanelli et al. [15] have recently reported that the adoption of a protocol of continuous infusion of levosimendan and amiodarone (starting at the commencement of surgery) has led to a marked reduction in the use of an IABP in patients with ischaemic cardiomyopathy who underwent surgical left ventricular (LV) restoration (predominantly surgical myocardial revascularization and/or mitral valve repair). Only two of 24 patients (8%) who received levosimendan required implantation of an IABP, compared with 11 of 38 patients (29%) who did not receive levosimendan (*p* = 0.018).

#### Valve surgery studies

Sheng and colleagues [16] have reported a prospective study of levosimendan conducted among 185 patients undergoing conventional valve replacement. In half of these patients, levosimendan was initiated immediately after ICU admission and added to standard care as a bolus dose of 10 μg/kg over 10 min, followed by infusion at a rate of 0.1–0.2 μg/kg/min over ≈24 h. Control patients received standard care plus placebo.

Levosimendan administration was associated with enhanced CO and LVEF at days 1, 3, and 7 after surgery (*p* < 0.05 for all versus control), and with a significant reduction in brain natriuretic peptide (BNP) (*p* < 0.001). It also significantly delayed the need for vasoactive drugs (dopamine and epinephrine) and reduced the dosages of those drugs used, as well as shortening the average length of ICU stay and reducing the incidence of postoperative adverse events, notably low CO (zero cases versus five; *p* = 0.023) and renal insufficiency (one case versus seven; *p* = 0.029).

#### Grown-up congenital heart disease studies

One study in grown-up congenital heart disease (GUCH) has been identified since 2019. In that retrospective work, Mauriat and colleagues compared outcomes in 87 patients undergoing cardiac surgery who received postoperative levosimendan (0.2 μg/kg/min/24 h) with those in 117 patients who received milrinone (0.5–1.0 μg/kg/min) [17]. Patients in either group could also receive low-dose (0.02–0.05 μg/kg/min) epinephrine or norepinephrine, as required. The vasoactive and inotropic score (VIS) was calculated for the first four postoperative days.

The primary outcome was the duration of mechanical ventilation (MV) because only haemodynamically stable patients without significant organ dysfunction were eligible to be extubated. Supplementary outcomes included epinephrine requirement, requirement for renal replacement therapy, and duration of ICU stay.

Patients in the levosimendan group had higher preoperative risk scores than the controls and a higher prevalence of left and right ventricular failure (RVF). After adjustment by propensity score weighting, however, levosimendan-treated patients had shorter durations of MV (average treatment effect –37.59 h; interquartile range [IQR] –138.85 to –19.13 h; *p* = 0.01) and ICU length of stay (average treatment effect –3.11 days; IQR –10.03 to –1.48 days; *p* = 0.009) plus a lower number of days of additional epinephrine application.

GUCH is characterized by a high prevalence of RVF and PH, and the pharmacological effects of levosimendan are relevant in that context. Importantly, although GUCH patients are at high risk of developing arrhythmia due to their underlying pathology, there was no additional morbidity due to arrhythmia in the levosimendan-treated patients when compared with the others. Other studies of levosimendan in RVF and PH are considered later in this review.

### Experts’ assessment

We recognize that, in cardiac surgery, levosimendan has been recommended in patients with LVEF < 35% undergoing CABG as a pre-emptive strategy to prevent LCOS [18, 19]. However, in the present update on its use in the ICU we focus on peri- and post-surgical use of levosimendan.

In a meta-analysis of 40 RCTs (*N* = 4246) of levosimendan versus any form of control therapy published in 2017, Putzu et al. concluded that there was “not enough high-quality evidence to neither support nor discourage the systematic use of levosimendan in cardiac surgery” [20]. Responses to the work of Putzu et al. included an alternative analysis by Jaguszewski and colleagues [21] who, limiting themselves to five studies of levosimendan versus dobutamine, demonstrated that levosimendan use was associated with lower in-hospital mortality, shorter average length of hospital stay, and lower risk of adverse events (specifically perioperative myocardial infarction and arrhythmias). Jaguszewski et al. profiled their response as demonstrating the hazards of over-aggregating heterogeneous data and what they described as the “urgent need to specify patient subpopulations who might truly benefit from levosimendan administration”.

In this context we note also the report of Woehrle et al. [22], who have documented a strategy of using low-dose levosimendan in the surgical setting. They propose, with some supporting evidence, that patients with preoperatively diagnosed LVEF ≤ 40% should receive 1.25 mg of levosimendan after induction of anaesthesia instead of the usual 12.5-mg dose. After surgery, administration of low-dose levosimendan may be repeated to attain or reach cardiovascular stability. In those authors’ cohort of 183 patients, a cumulative dosage of ≤ 5 mg levosimendan was considered sufficient for three-quarters of cases, with reductions (relative to findings from published control cohorts) in maximum doses of epinephrine, the incidence of atrial fibrillation, and 30-day mortality.

Measures to optimize not only patient selection but the dosing of levosimendan are pertinent to the wider issues of how, when, and for whom levosimendan is used in postoperative care but the study of Woehrle et al., while conceptually engaging, is too small to resolve the matter, beyond perhaps re-emphasizing the need for a highly individual approach tailored to the specific needs of individual patients. Prima facie these indications of benefit from low-dose levosimendan are at variance with the experience of the CHEETAH study [23], in which low dose (mean infusion rate 0.07 μg/kg/min) levosimendan had no impact on designated endpoints, and from LICORN [24], which provided no indications of efficacy from preoperative use of levosimendan. Issues of case-mix and differences in timing of administration (plus the experimental nature of the endpoints used in LICORN) make direct comparisons of these trials infeasible and very possibly unhelpful. These points have been scrutinized by Guarracino et al. [19] in an opinion paper on the subject.

Viewed from that perspective, the works of Weber et al. and van Diepen et al. support the view that future studies should focus on:

- early administration of levosimendan
- patients with established low LVEF (≤ 35–40%) and
- patients undergoing isolated CABG surgery.

A full elaboration of the criteria for such studies requires a further detailed interrogation of the available data which is beyond the scope of this commentary.

Conversely, the work of Sheng et al. [16] encourages the view that ICU-initiated use of levosimendan may be advantageous to patients recovering from cardiac valve surgery. This observation suggests a further and additional area of use for levosimendan, while at the same time illustrating the interplay between surgical status and drug timing.

Matching the appropriate type of patient to the appropriately timed, appropriately dosed intervention remains a challenge for the design of adequately powered, informative clinical trials. The observations of Kocabeyoglu et al. [25] on the postoperative optimization of left ventricular assist device implantation by levosimendan is a further example of interesting potential applications that merit exploration, but which might be difficult to substantiate without very close monitoring of multiple potential confounding influences.

## Septic shock

### Recent meta-analyses

Liu and colleagues [26] performed a meta-analysis restricted to RCTs that compared levosimendan with dobutamine. Six such studies, involving a total of 192 patients, were identified. These patients were characterized as experiencing sepsis-induced cardiac dysfunction. The terms “shock” and “septic shock” were not used in the published search strategy but, in discussion of their findings, the authors asserted that “*septic shock patients* [our emphasis] were randomized to receive either levosimendan (0.2μg/kg/min) or dobutamine (5μg/kg/min) after achieving normovolaemia and a mean arterial pressure of at least 65 mmHg in all the included studies”. Five of the six studies identified compared the effect of a 24-h infusion of levosimendan (0.2 μg/kg/min) with a similar duration of dobutamine (5 μg/kg/min); the sixth study compared the impact of adding 24 h of levosimendan infusion (also at a dose of 0.2 μg/kg/min) to patients who had received dobutamine 5 μg/kg/min for 48 h.

**Fig. 1:**
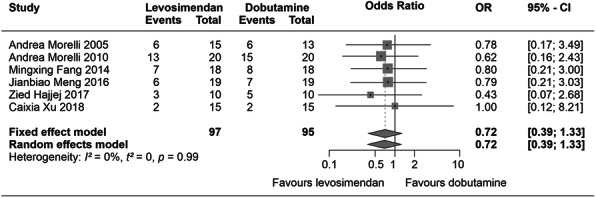
Meta-analysis of RCTs comparing levosimendan with dobutamine in patients experiencing sepsis-induced cardiac dysfunction. Forest plot of mortality. OR, odds ratio; CI, confidence interval. *I*^2^ = 0%, *p* = 0.99, fixed effects, OR: 0.72, 95% CnI: 0.39–1.33. Figure drawn freely from the data reported by Liu et al. [26]

Compared with dobutamine, levosimendan treatment was associated with statistically significant improvements in a range of cardiac function indices, including ΔCI (random-effects model standardized mean difference [REFSMD] 0.90; 95% CnI 0.20–1.60; *p* < 0.01) and left ventricular stroke work index (ΔLVSWI) (REFSMD 1.56; 95% CnI 0.90–2.21; *p* = 0.04). There was also a significant reduction in blood lactate (REFSMD − 0.79; 95% CnI − 1.33 to − 0.25; *p* < 0.01) at 24 h but no significant enhancement of LVEF (REFSMD 0.77; 95% CnI 0.41–1.12; *p* = 0.42). Substantial heterogeneity was apparent in the data but sensitivity analysis supported some tangible effect of levosimendan on these indices. Mortality trends in the majority of the studies narrowly favoured levosimendan but this effect did not reach the threshold of statistical significance either in individual studies or in aggregate (Fig. 1). It should be noted that these data were derived from the longest-reported dates in each study and that these varied from death during ICU stay to death at 28-day follow-up.

The authors of this analysis considered themselves constrained by a small and methodologically uneven dataset. They also stressed that “Considering severe sepsis and septic shock part of the same entity could have led to heterogeneity” and cautioned that the improvement in serum lactate levels could not confidently be ascribed to any action of levosimendan because of the confounding influence of fluid resuscitation (average fluid input was higher in levosimendan-treated patients than in controls).

Randomized controlled studies of levosimendan versus either inotropic drugs or placebo for the treatment of sepsis or septic shock were included in the second recent meta-analysis. Feng and colleagues [27] identified 20 such trials that enrolled an aggregate of 1467 patients (levosimendan, *n* = 738; control, *n* = 729). Findings very similar to those of Liu et al. were documented, with indications of improved cardiac function in the levosimendan group, plus reduction in serum lactate, but no robust impact on mortality.

Limitations of this analysis include the fact that the distributions of “other inotropes” and “placebo” were not reported. In addition, the LEOPARDS trial [28] contributed 50% of the weight of this meta-analysis, dominating the mortality finding. Further explorations from the LEOPARDS study have recently come into the public domain with the appearance of an analysis of mortality outcomes in patients who had biochemical indications of cardiac dysfunction (essentially, raised cardiac troponin I and N-terminal [NT]-proBNP). In all, 442 of the 516 patients enrolled contributed to this sub-analysis, which conformed to the primary analysis in finding no effect of levosimendan on mortality [29].

### Recent clinical trials

No clinical trials were identified within our prespecified time range.

### Expertsʼ assessment

The rationale for levosimendan as an important addition to medical interventions in sepsis was much enhanced in 2015, when a meta-analysis by Zangrillo et al. [30] appeared to show that, in addition to the cardiovascular and blood chemistry effects reiterated in the more recent studies outlined above, levosimendan also reduced mortality (47% vs 61%; *p* = 0.03; number needed to treat of seven). This striking effect, however, emerged from a small cohort of patients (*n* = 246 in seven trials) and the large LEOPARDS trial [28] provided no evidence of any effect on mortality of any of ICU discharge, hospital discharge, or mortality at 28 days.

LEOPARDS also demonstrated no significant benefit of levosimendan on the study primary endpoint of mean daily Sequential Organ Failure Assessment (SOFA) score or on any of the SOFA score domains. As noted above, a recent additional analysis from that same study has provided no indication of a survival benefit in patients deemed likely to have sepsis-related cardiovascular dysfunction at baseline on the basis of blood chemistry criteria. We highlight, however, that in fluid-resuscitated sepsis (even when hyperdynamic), BNP is increased because of the dilatation of the atria caused by the rapid administration of fluid; the elevation of BNP is not unequivocally related to septic cardiomyopathy. For this reason, the re-analysis of LEOPARDS data by Antcliffe et al. [29] could not be expected to show better results. We note, inter alia, that the authors of that sub-analysis also asserted that they found no evidence of reductions in any inflammatory mediators in patients treated with levosimendan, seemingly rebutting various ingenious hypotheses in this regard.

There are important mitigating factors to consider. Firstly, the inclusion criteria in the LEOPARDS trial did not specifically focus on septic patients with myocardial dysfunction or even cardiomyopathy. Instead, the inclusion was primarily based on the need for a vasopressor. Additionally, cardiac function was not adequately addressed, either by echocardiography or by extended haemodynamic monitoring. Consequently, the authors of a comment on the LEOPARDS study concluded [31] that “this trial does not add more than the information that patients with sepsis who presented with reduced systemic vascular resistance but without clinical signs of myocardial dysfunction did not benefit from treatment with a potent inodilator drug”.

Secondly, it may be noted that there may be a case for favouring levosimendan over dobutamine in sepsis patients who require inotropic support. Liu et al. [26] conjecture that downregulation of beta-receptors in sepsis may attenuate the increase in CI caused by dobutamine and suggest that, while the dose of dobutamine used in the studies they identified (5 μg/kg/min) may therefore be insufficient in this setting, any higher dose may come with an increased risk of cardiac arrhythmias. We also note data indicating a survival benefit from beta-blockers in sepsis [32, 33]; this implies an adverse impact of beta-adrenergic activity on survival in sepsis that might be addressed (at least in part) by avoiding dobutamine and reducing noradrenaline in favour of levosimendan.

This conjecture remains to be tested and it has to be acknowledged that levosimendan itself was associated with a higher incidence of supraventricular arrhythmias in LEOPARDS. However, as fewer than 10% of the study population received dobutamine, those data are unlikely to be a fully informative comparison of the two drugs. It should also be noted that, although LEOPARDS reported no significant treatment effect on a pre-specified subset of patients with baseline low CI, that subset was very likely too small (*n* = 52) to detect such a difference. The data reported by Antcliffe et al. [29], although prima facie inconclusive, similarly do not offer insights into the relative effects of levosimendan and dobutamine.

For the moment, the use of levosimendan has to be substantially guided by the advice of the Surviving Sepsis Campaign (SSC) [34], which states that “For adults with septic shock and cardiac dysfunction with persistent hypoperfusion despite adequate volume status and arterial blood pressure, we suggest against using levosimendan (weak, low-quality evidence)” with the caveat that recommendations cannot replace a clinician’s decision-making for individual cases. On the other hand, the guidelines do not argue against the use of levosimendan in septic shock patients with LCOS documented by echocardiography, pulmonary artery catheter, or pulse index continuous cardiac output.

One route towards a fuller understanding of the place of levosimendan in sepsis treatment may lie through highly granular exploration in small numbers of patients, as currently exemplified by the Levosimendan and Global Longitudinal Strain Assessment in Sepsis (GLASSES-1) observational study (NCT 04141410). Readers are referred to the full published protocol of this study to appreciate its details [35].

Other initiatives that might help to position levosimendan in sepsis include the use of methodologies such as side-stream dark-field imaging of the microcirculation [36] to investigate the relation between systemic haemodynamic effects of levosimendan and changes in the microcirculation that might be relevant to organ perfusion in sepsis. We know of no such studies currently in progress or development.

Finally, we agree with the conclusions of the recent editorial by Vincent and co-authors [37] on the need to equilibrate the SSC guidelines with individualized care. In the words of the authors, “The large majority of randomized, controlled trials performed over the last three decades in intensive care medicine, including those in sepsis, have shown no significant beneficial effect of the tested intervention on outcomes. At face value, this may simply suggest that the myriad of interventions that have been tested are all ineffective. However, it is more likely that subsets of patients who benefit from specific treatments have yet to be identified. The often broad patient inclusion criteria could easily lead to dilution of positive findings by non-responders, or to positive effects in some patients being offset by harm in others”. (See Santacruz et al. [38] and Vincent and Sakr [39] for additional perspectives on these issues.)

Concisely, septic cardiomyopathy is a complex diagnosis that can lead to too much variability in the inclusion criteria of studies, paving the way to inconclusive results [40].

## Weaning from mechanical ventilation

### Recent meta-analyses

No meta-analyses were identified within our prespecified time range.

### Recent clinical trials

A pilot study of the effect of levosimendan in weaning from MV in patients with LV dysfunction has been reported recently by Kaltsi et al. [41]. In this prospective, single-centre work, 19 patients with a mean age of 73 ± 8 years underwent an initial spontaneous breathing trial (SBT). These were critically ill patients, mechanically ventilated because of acute respiratory failure of various aetiologies (six surgical, 13 medical), who had difficult or prolonged weaning from MV and LV systolic dysfunction, defined as LVEF < 50%. Patients had been supported by MV for 7–56 days (median 11 days) and had repeatedly failed weaning trials.

**Fig. 2:**
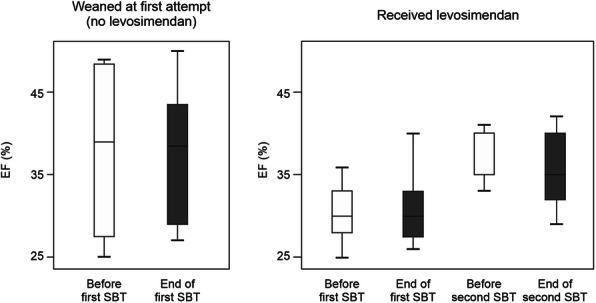
Effect of levosimendan in weaning from mechanical ventilation in patients with left ventricular dysfunction. Left ventricular ejection fraction (LVEF) before the start (white boxes) and at the end (grey boxes) of spontaneous weaning trials in successfully weaned patients (**left**) and in those who failed and received levosimendan (**right**). LVEF increased after levosimendan administration. Figure drawn freely from the data reported by Kaltsi et al. [40]. SBT, spontaneous breathing trial

Eight patients were successfully weaned on the first SBT. The remaining 11 were re-ventilated and received levosimendan as a 24-h i.v. infusion at a dose of 0.1–0.2 μg/kg/min according to haemodynamic effect. No loading dose was used, and no other medication was added to the existing treatments. A second SBT was attempted within 24 h of completing the levosimendan infusion. On that occasion, nine of the 11 patients were successfully weaned. This clinical success was accompanied by a significant increase in mean LVEF and reduction of filling pressure (Fig. 2). LV relaxation was also enhanced, suggesting a treatment effect on both systolic and diastolic ventricular function.

Studies by a Dutch–Canadian research collaboration demonstrated in vitro that levosimendan improved the contractile performance of respiratory muscle tissue from elective surgery patients without comorbidities and patients with chronic obstructive pulmonary disease. Moreover, they demonstrated that levosimendan improves in vivo contractile efficiency of the diaphragm in healthy subjects [42, 43]. In their most recent investigation of this phenomenon, these investigators administered levosimendan (0.2 μg/kg/min) or placebo for 5 h to a total of 39 mechanically ventilated ICU patients to see if this intervention helped to overcome ICU-acquired weakness of the respiratory muscles, particularly those of the diaphragm. Tidal volume and minute ventilation were higher after levosimendan administration (by 11% and 21%, respectively) but, in apparent contrast to earlier findings, there were no indications of enhanced neuromechanical efficiency of the diaphragm or enhancement of contractile force. Levosimendan was associated with a reduction in mean arterial blood pressure and an increase in heart rate, consistent with its established haemodynamic profile.

### Experts’ assessment

The use of levosimendan as an aid to weaning rests in part on the premise that cardiovascular dysfunction arising from causes such as increased LV preload and afterload, as well as a decrease in LV compliance arising from the transition to negative intra-thoracic pressures, is a major factor in failure to wean. In addition, the activation of the sympathetic nervous system that accompanies a failed SBT militates, at least conceptually, against the use of exogenous catecholamines. Use of a non-adrenergic agent to optimize cardiac function before an SBT is thus an appealing idea made feasible by the availability of levosimendan [44], and for facilitating beta-blocker therapy according to heart failure guidelines.

The studies reviewed here were small and that of Kaltsi et al. included patients with notably heterogeneous pathologies. Lack of randomization or a well-defined control group also militates against emphatic claims of treatment efficacy. The trend of the clinical outcomes in these studies is, however, persuasive of the idea that levosimendan may be beneficial in these settings and some of the attendant physiological observations are instructive as to the possible bases for those findings, which may include reduction in the use of high-dose conventional inotropes, with their attendant hazards (although the use of such drugs may be a proxy and indicator of innate poor recoverability of myocardial function). These data are thus an encouragement to further research. The role of levosimendan in patients with ventilator weaning difficulties due to respiratory muscle weakness requires further clinical studies.

## Weaning from VA-ECMO

### Recent meta-analyses

The possible impact of levosimendan on success in weaning from VA-ECMO has been the subject of two recent meta-analyses, one by Burgos et al. [45] and the other by Kaddoura and colleagues [46]. Both drew on substantially the same dataset (all observational or non-randomized studies), the only discrepancy being the inclusion in one analysis of data from 63 patients from an observational study in France reported in abstract form only. Findings from these two meta-analyses may be aggregated and summarized in the following terms.

1. The use of levosimendan appears to be associated with a significant increase in the odds of successful weaning from VA-ECMO and also with a significant survival advantage. The first of these conclusions is based on three studies that each contributed ≈ 25% to the weight of the meta-analysis and may therefore be regarded as reasonably well founded. The mortality finding is heavily dependent on data from a single study that accounted for > 50% of the weight of the meta-analysis and must therefore be regarded as a less secure finding.
2. Multiple and extensive sources of bias and possible confounding exist in the source studies. Therefore, findings and conclusions from these meta-analyses should only be regarded as a basis for prospective studies to fully elucidate this matter.

Similar conclusions on both endpoints were reached by Yang et al. [47], who noted that their finding on survival was resilient in one-way sensitivity analysis.

Further insights into this matter are provided by the meta-analysis of Luo et al. [48] which draws on data from 2274 patients in 18 studies (RCTs or observational studies published in either Chinese or English). Pooled analysis from all 18 studies indicated that levosimendan improved MV/ECMO weaning (*n* = 2274; OR 2.32; 95% CnI 1.60–3.36; *p* < 0.00001 vs control), albeit with high heterogeneity (*I*^2^ = 68%) and a general rating of evidence quality as low to moderate. Subgroup analyses confirmed significantly better rates of weaning success (*p* ≤ 0.004) for levosimendan versus controls in patients with low LVEF and MV (four studies), patients with ECMO after cardiac surgery (four studies), or patients with ECMO plus CS (five studies), but no evidence of the effect on weaning from MV (four studies; OR 2.25; 95% CnI 0.72–7.25). It may be noted that the lack of effect in this last category owed much to the influence on the analysis of data from the LEOPARDS trial, already discussed in the sepsis section of this commentary.

Mortality, assessed as a secondary endpoint (six studies; 596 patients), was lower in levosimendan-treated ECMO patients than controls (OR 0.66; 95% CnI 0.53–0.81; *p* < 0.0001) but was not similarly reduced in mechanically ventilated patients.

### Recent clinical trials

Less affirmative findings accrued from the observational study by Guilherme and colleagues [49] in adult patients with refractory CS. Comparison of outcomes from 48 patients treated with levosimendan (0.1 μg/kg/min for 1 h, then 0.1–0.2 μg/kg/min for another 23 h) and 78 matched controls identified a small but non-significant reduction in the VA-ECMO weaning failure rate with levosimendan (29.1% vs 35.4%; OR 0.69; 95% CnI 0.25–1.88). No benefit of levosimendan was seen on mortality at 28 days or at 6 months.

Alonso-Fernandez-Gatta et al. [50] reported that in a retrospective analysis of 123 VA-ECMO cases at a single centre, 23 patients received a continuous infusion of 0.05–0.1 μg/kg/min levosimendan and that this treatment was associated with a higher (although not significantly different) weaning rate of 61% (vs 44% in the patients not receiving levosimendan), notwithstanding that levosimendan was administered more frequently in patients with a lower LVEF at the time VA-ECMO was commenced (mean 18% vs 31%). There was also a survival gain with levosimendan (52%, vs 36% at discharge) but, like the weaning rate difference, this finding was not statistically significant.

Also noteworthy in this context is work by Mahesh et al. [51] on a novel strategy for improved outcomes of ECMO as a treatment for refractory post-cardiotomy CS in the current era, in which the authors give a refreshing new perspective and suggest a new management algorithm including levosimendan [0.2 μg/kg/min for 24 h].

### Experts’ assessment

Outcomes in weaning from VA-ECMO can differ materially depending on the reason for commencing VA-ECMO in the first place. (Potential for myocardial recovery as inferred from the recorded indication(s) for starting VA-ECMO featured in the propensity score developed by Guilherne et al.) The evidence so far available suggests that levosimendan may be more efficacious in cases of postoperative LCOS or refractory cardiac shock than in cases of refractory cardiac arrest, but these conjectures require substantiation in a controlled trial. The duration of VA-ECMO also exerts an influence on the eventual success of weaning [52, 53] and the interplay between that and the optimal timing and dosing of levosimendan in this scenario also requires further investigation. No adverse impact of levosimendan on VA-ECMO-related complications, including but not limited to bleeding or neurological incidents, has been noted but further assurance on this point is desirable.

Prominent among initiatives in this area is the WEANILEVO trial (NCT 04158674). Conducted at seven French tertiary cardiovascular ICUs with experience in double peripheral venous and arterial cannulation for extracorporeal membrane oxygenation (VA-VA-ECMO), this randomized, prospective, placebo-controlled, multicentre, double-blind, parallel-group trial aims to evaluate the efficacy of levosimendan (0.2 μg/kg/min for 24 h) in reducing VA-VA-ECMO weaning failure in adult patients with AHF. Weaning criteria, set out at length in the published protocol [54], include arterial lactate ≤ 2 mmol/l, right ventricular end-diastolic diameter < 35 mm, and combined fraction of inspired oxygen for VA-ECMO and ventilator < 80%.

The primary endpoint of WEANILEVO is VA-VA-ECMO weaning failure or death within 7 days of VA-VA-ECMO weaning. This is specifically chosen to cover the period of time during which levosimendan or its active metabolite can be expected to be present in the body at therapeutically relevant concentrations. Secondary endpoints include the need for long-term mechanical circulatory assistance, heart transplant within 30 days of weaning, and 30-day all-cause mortality. The study design predicates recruitment of 180 patients, the first of whom was randomized in February 2020. Follow-up is scheduled for completion in 2024.

**Fig. 3:**
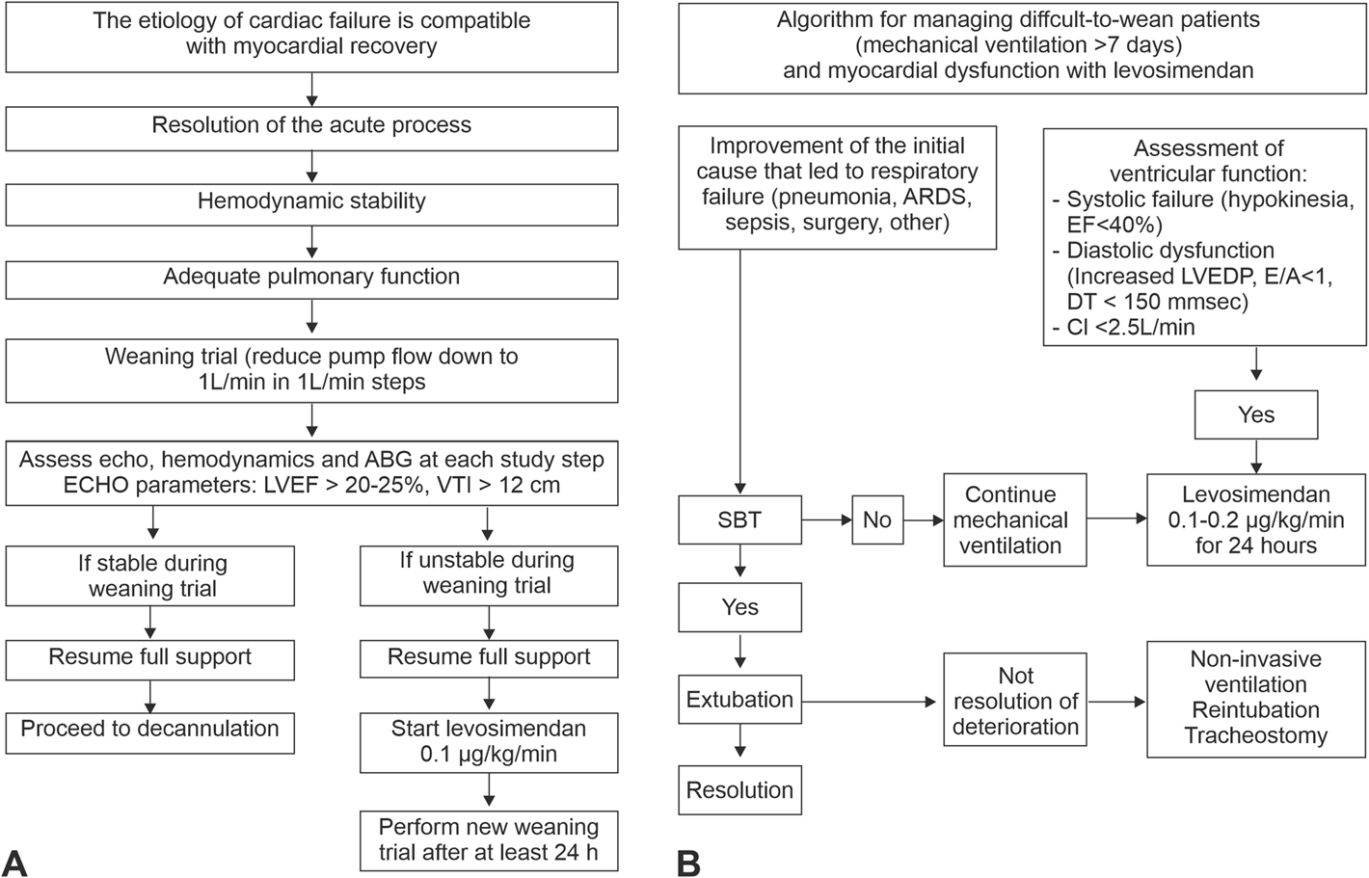
Algorithms for weaning from **a** extracorporeal membrane oxygenation and **b** mechanical ventilation which include levosimendan. Figure drawn freely from Sangalli et al. [55] E/A, ratio of early to late (atrial) peak blood flow during diastole; DT, deceleration time; LVEDP, left ventricular end-diastolic pressure

Pending the results of WEANILEVO and other investigations, the algorithms recently advocated by Sangalli et al. [55] may provide a starting point for pragmatic discussions of how to locate levosimendan in the weaning pathway (Fig. 3). Meanwhile, we agree with the recommendations in the ESC heart failure guidelines that “Levosimendan may be considered in patients with cardiac dysfunction to facilitate weaning from MV as well as VA-ECMO in conjunction with standard heart failure therapy” [56].

## Cardiogenic shock

### Recent meta-analysis

The most notable recent addition to the literature on the role of levosimendan in CS is the 2020 update of a Cochrane Collaboration systematic review [57]. The central finding of that review is that the current dataset is wholly inadequate to guide practice, with the authors reporting that there are “no convincing data supporting *any* [our emphasis] specific inotropic or vasodilating therapy to reduce mortality in haemodynamically unstable patients with CS or LCOS”.

This conclusion was derived from analysis of 19 RCTs that enrolled patients (*N* = 2385) with acute myocardial infarction (AMI), heart failure, or cardiac surgery complicated by either CS or LCOS. Studies in patients with sepsis were not included. Data were also accrued from three trials ongoing at the time of the analysis. Overall, the data were assessed as being uniformly of low or very low quality with extensive potential for biases and correspondingly low reliability of all effect estimates on short- or long-term mortality.

Eleven distinct analyses were undertaken, including levosimendan versus dobutamine, enoximone, or placebo. Findings in detail for those three analyses were as follows:

- Levosimendan versus dobutamine: Short-term mortality (*n* = 1701): relative risk (RR) 0.60, 95% CnI 0.36–1.03. Long-term mortality (*n* = 1591): RR 0.84, 95% CnI 0.63–1.13. Low-quality evidence for both outcomes.
- Levosimendan versus placebo: Short-term mortality: no data. Long-term mortality (*n* = 55): RR 0.55, 95% CnI 0.16–1.90; very-low-quality evidence.
- Levosimendan versus enoximone: Short-term mortality (*n* = 32): RR 0.50, 95% CnI 0.22–1.14; very-low-quality evidence. Long-term mortality: no data.

These data are supplemented by the work of Liao et al. [58], who conducted a ‘reticular network meta-analysis’ of the safety of an array of possible therapies for CS, including levosimendan, dobutamine, milrinone, dopamine, adrenaline, noradrenaline, and recombinant human brain natriuretic peptide (RHBNP). Based on 28 studies that included 1806 patients, Liao et al. concluded that milrinone was the preferred agent based on its efficacy and potential for side effects, with levosimendan and RHBNP as other strong candidates. Sources of bias in many of the source reports were noted but not described in detail. The particular nature of the methodology of this analysis limits its applicability to clinical decision-making for individual cases.

In their meta-analysis of eight placebo-controlled trials of levosimendan in AMI, Tumminello et al. [59] found a beneficial effect of levosimendan on acute and long-term mortality of patients affected by AMI. Levosimendan may thus also prevent the development of myocardial insufficiency in AMI.

### Recent clinical studies

Wang et al. [60] have reported on findings with levosimendan in 24 closely documented patients with low (one to four) Interagency Registry for Mechanically Assisted Circulatory Support (INTERMACS) scores. Eight of these patients had INTERMACS scores of one and had their circulation supported by mechanical interventions (mechanical circulatory support [MCS]). Those patients received a 24-h infusion of levosimendan (6–12 μg/kg/20 min then 0.1 μg/kg/min) immediately after MCS implantation; other patients commenced the same levosimendan regimen on admission to the ICU. The aetiology of CS varied in these patients, but all had evidence of renal insufficiency and lactate acidosis. Most of the patients had diagnoses of chronic heart disease and were already in receipt of optimal medical therapy where not contraindicated. All patients received inotropes (preferably dopamine, otherwise dobutamine and/or norepinephrine) to support haemodynamics.

Systemic blood pressure and heart rate were unchanged during levosimendan infusion but maintenance of these indices required significantly less aggregate use of other inotropes (*p* = 0.024). Daily urine output increased at 72 h after levosimendan administration (2142.4 ± 429.8 ml, vs 1360 ± 385.4 ml at baseline; *p* = 0.018). BNP levels were also significantly reduced at that time (*p* = 0.007) and several biochemical markers of hepatic status showed nominally advantageous but non-significant changes.

Two months after levosimendan treatment, LVEF was significantly improved (35.9 ± 13.4%, vs 22.4 ± 8.1% at baseline; *p* = 0.001), LV volumes were reduced, and health-related quality of life, also assessed at 2 months using the Kansas City Cardiomyopathy Questionnaire (KCCQ), was significantly enhanced (*p* < 0.001). Five of the eight patients who needed MCS at the outset were successfully bridged to recovery; a sixth was eventually bridged to heart transplantation, and the other two died of multiple organ failure. Two of the patients with INTERMACS scores of two were bridged to transplantation and four died of CS, while the 10 patients with INTERMACS scores of three or four initially were described as having “uneventful recovery to discharge”.

In other recent granular research, Grossini and colleagues [61] have reported data that they interpret as showing that levosimendan can regulate oxidant/antioxidant balance in CS patients and have suggested that modulation of oxidative status at a mitochondrial level may contribute to a possible cardio-protective role for levosimendan. This interesting line of enquiry is so far based on observations from four patients, however, and is thus very much in need of elaboration.

Guilherme et al. [49] have examined the use of levosimendan to mitigate VA-ECMO weaning failure in CS in an observational single-centre study (*N* = 200). This study is considered earlier in this review in our assessment of levosimendan as an aid to weaning.

For the sake of completeness, we cite here a recent case report by Fox and colleagues [62] in which synergistic effects of levosimendan and convalescent plasma were described as bailout strategy in acute CS in COVID-19. We must emphasize, however, that no prospective trial has explored the impact (if any) of levosimendan in COVID-19-associated CS.

### Experts’ assessment

Despite their own assessment of the limitations of the database, the authors of the 2020 Cochrane systematic review [57] were guardedly encouraging about the use of levosimendan in CS, stating: “In terms of haemodynamic improvements, levosimendan may be useful for haemodynamic stabilization but there are still major concerns as to whether these improvements translate into prognostic benefits. This is particularly true in the settings when inotropes need to be combined with vasopressors. Given the favourable safety profile, levosimendan may be considered for therapeutic escalation.”

These views are broadly endorsed by other recent commentaries. Thus, for example, the S-T segment elevation myocardial infarction (STEMI) guidelines prepared by Ibanez et al. [63] conclude that levosimendan “may be considered an alternative, especially for patients on chronic beta-blocker therapy, because its inotropic effect is independent of beta-adrenergic stimulation. Phosphodiesterase III inhibitors are not recommended in STEMI patients”. On the same note, Werdan et al. [64] recommend that “In patients refractory to catecholamines, levosimendan should be preferred over phosphodiesterase III inhibitors. In light of potentially serious adverse events, caution is required with regard to catecholamine administration; especially in clinically ‘marginally’ stable ICS [infarction-related cardiogenic shock] patients, catecholamine administration is not generally required”. Similarly, De Backer et al. [65], despite favouring dobutamine as the first-line agent in CS, endorse levosimendan as “an excellent alternative or additional agent in cases not responding to dobutamine”. Finally, Shabana and colleagues [66] go further and propose a combination of levosimendan and norepinephrine as the “most effective management option in CS”. This view rests substantially on the assessment of the favourable safety profile of levosimendan but all authors also stress that choice of therapy should be “individualized and based on the hemodynamic response”. We agree with these conclusions.

## Takotsubo syndrome

### Recent meta-analysis

Jaguszewski et al. [67] have recently examined the effect of levosimendan in TTS in a pooled analysis of eight studies contributing 272 patients, 135 of whom received levosimendan. These patients had an average age of 70 years and the predominance of female patients (≈ 70%) reflected the predominance of TTS among females in general populations. All patients were classified at the outset as being in New York Heart Association (NYHA) class IV, with initial LVEF ≈ 30%. There was extensive use of diuretics (≈ 72%) and rather less use of angiotensin-targeting medications (≈ 38%) and beta-blockers (≈ 15%).

Assessed at discharge or after 30 days, use of levosimendan was associated with higher LVEF (*p* < 0.01), more rapid recovery of LVEF to > 50% (5.1 ± 1.6 vs 8.2 ± 2.4 days; *p* < 0.001), and shorter average duration of hospital stay (9.4 ± 1.7 vs 14.3 ± 1.5 days; *p* < 0.001). These differences were based, however, on small patient numbers (*N* = 42). Mortality, based on a larger subset (*N* = 222), was not significantly different, perhaps because of the low overall death rates in the two groups (3/122 [2.5%] vs 8/100 [8.0%]; *p* < 0.07).

In separate research based on the International Takotsubo Registry, the enhanced recovery of LVEF associated with levosimendan use was regarded by the authors of this analysis as encouraging expectations of a survival benefit from levosimendan in TTS, but the lack of an overall and robust effect on mortality led them to conclude that additional prospective studies are needed to build on these data [68].

### Recent clinical studies

The only randomized double-blind study identified in this meta-analysis was the work of Guo et al. [69] in 200 elderly Chinese TTS patients. This was by far the largest data source for the meta-analysis and exerted a powerful influence on the overall findings. Closer attention to that study is therefore appropriate. Levosimendan was given as a continuous i.v. infusion at a rate of 0.1 μg/kg/min for 24 h in addition to unspecified “regular treatment”; no loading dose was used. Most interestingly, at 30 days follow-up, there was a significant survival advantage with levosimendan (one death versus eight; *p* < 0.041); no further deaths were recorded to 180 days (still *p* < 0.041). All patients in this study were aged ≥ 65 years (average 71 years), so the generalizability of this survival finding cannot be taken for granted. This work has been critiqued by Finsterer and Stoellberger, [70] who noted deficits in the reporting of comorbidities, specific presentations of TTS, and co-medication in the two study groups and emphasized the strong desirability for a control group that received no cardiac treatments (reflecting the fact that many TTS patients recover without intervention). The Spanish National Registry on Takotsubo syndrome (RETAKO) offers an additional perspective on this issue, documenting a profound adverse mortality impact of concomitant CS in TTS, possibly through being an identifier of what the RETAKO authors describe as “a masked heart failure phenotype with increased vulnerability to catecholamine-mediated myocardial stunning” [71].

Of further note in this context is recent work by Trinh-Duc et al. [72] that elegantly supports the rationale of using levosimendan instead of dobutamine or phosphodiesterase-III inhibitors to treat TTS induced by a subarachnoid haemorrhage: saving the brain from disastrous vasospasms while protecting the heart from excessive catecholamines and promoting recovery of faster contractile function. This is a development of a concept introduced in previous publications [73, 74] but is still at too early a stage of assessment to be commented on further in this review.

### Experts’ assessment

Emergence of TTS is often connected with major physical or emotional stress and an associated surge in plasma catecholamine levels is thought to be part of the precipitating pathophysiology. This theory creates an a priori presumption against the use of adrenergic agents as part of the treatment response, for which retrospective analysis of outcomes at a single centre in Mannheim, Germany provides some evidential basis [75]. As a non-adrenergic inodilator, levosimendan may therefore be of special utility in this scenario.

There are encouraging signs in the investigations we have reviewed but the limitations of the database for levosimendan in TTS will nevertheless be evident. The pre-eminence in recent clinical research of a study that has been criticized for various potential methodological shortcomings [70] means that that study and any meta-analyses dominated by it cannot securely demonstrate the utility of levosimendan in this application. Additional well-configured trials in this area are therefore needed.

## In the presence of renal dysfunction/failure

### Recent meta-analyses

A series of meta-analyses have recently explored the impact of levosimendan on renal function. Long et al. [76] undertook a conventional analysis of 28 trials in patients with LV dysfunction (*N* = 5069) and concluded that the use of levosimendan was associated with improvements in various signifiers of renal function, including serum creatinine (*p* = 0.005), the risk of acute renal failure (*p* = 0.017), glomerular filtration rate (GFR) (*p* = 0.092), and urine output (*p* = 0.024) but noted that these findings were derived from evidence that was regarded as very low to moderate in quality. Their conclusion that “Levosimendan might improve renal function of patients with left ventricular dysfunction” was thus carefully circumscribed.

Chen et al. [77] produced a network meta-analysis in March 2020 with a report on renal dysfunction in patients undergoing cardiac surgery and later added a second report based on 37 published studies that included 4957 patients [78], most of whom were classified as having advanced heart failure. A feature of this research was its use of *P*-scores to provide relative rankings of the efficacy of interventions. (A *P*-score reflects the degree to which any given treatment is superior to other treatments. It can range from 0% [relatively very poor results] to 100% [relatively very good results].)

In the cardiac surgery network analysis (29 RCTs), mortality data were reported for 3641 patients and data regarding the incidence of AKI for 2678. Levosimendan significantly reduced mortality compared with placebo (OR 0.74; 95% CnI 0.56–0.97), while dobutamine increased mortality compared with placebo. Levosimendan was also significantly associated with a lower incidence of AKI compared with placebo (OR 0.61; 95% CnI 0.45–0.82).

**Fig. 4:**
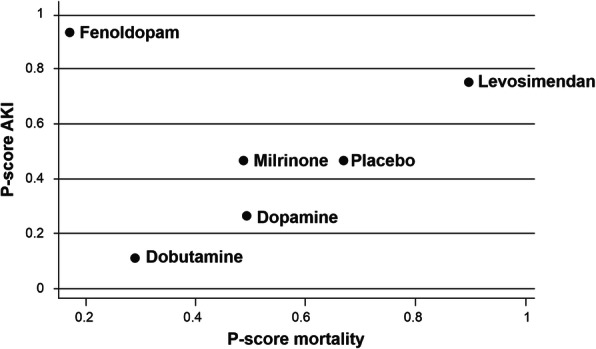
Mortality data and the incidence of acute kidney injury (AKI) reported for 3641 patients in a cardiac surgery network analysis. P-scores ranking plot showing mortality and AKI associated with interventions. Treatments with better efficacy should be in the upper-right-hand corner of the graph. Figure drawn freely from the data reported by Chen et al. [77]

In the *P*-score ranking analysis of mortality reduction, levosimendan outperformed a range of other inotropic agents (dobutamine, dopamine, fenoldopam, and milrinone). Although fenoldopam exhibited a slightly better *P*-score than levosimendan (and a substantially better score than other comparators) in AKI, its use seemed to be associated with an increase in mortality risk. Hence, when *P*-scores for mortality and AKI were compared graphically, levosimendan emerged as conspicuously the best performing of the interventions assessed (Fig. 4).

The use of levosimendan also significantly reduced ICU length of stay compared with placebo (mean difference: − 0.55; 95% CnI − 1.00 to − 0.09), whereas the use of dobutamine significantly increased ICU length of stay compared with all other inotropes or with control. Further analysis established that the survival benefit of levosimendan was seen in patients with initially low LV systolic function. (Patient numbers for preserved systolic function were too small to support any conclusion.) Timing of administration and type of surgery performed did not influence the response to treatment. These data were derived entirely from RCTs and were hence judged as being of reasonable quality by the authors, but they noted a moderate to high risk of bias arising primarily from a lack of blinding.

### Recent clinical trials

These aggregated findings are amplified by observations in a series of recent trials. Guerrero Orriach et al. [79] have conducted a 3-year study in cardiac surgery patients with LCOS. A total of 100 adult patients meeting pre-specified criteria for LCOS requiring inotropic support were recruited, of whom 50 received levosimendan (0.1 μg/kg/min/24 h to a target dose of 12.5 mg) and 50 received unspecified beta-agonist inotropes at undisclosed dosages. Thirty of the 100 patients had evidence of kidney failure at the time of diagnosis of LCOS (15 per group). Six of 15 patients in the beta-agonist group still had evidence of kidney failure at discharge, compared with none in the levosimendan group. Significant (*p* < 0.05) differences were observed at 48 h in creatinine levels, diuresis volumes, and AKI scores between patients treated with levosimendan and those treated with beta-agonists; quantitatively, those differences were especially marked between the 15 patients from the levosimendan group and the six beta-agonist-treated patients who still had kidney failure at discharge.

**Fig. 5:**
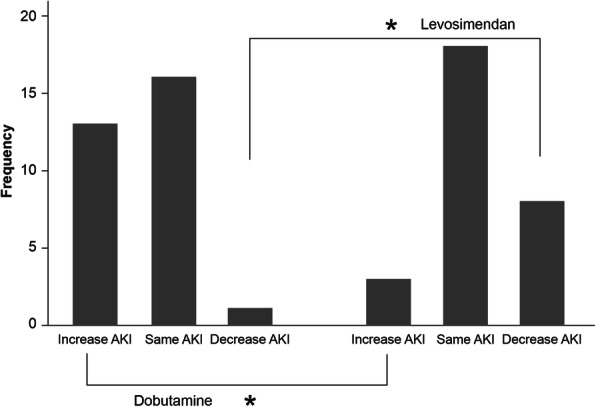
Variation of acute kidney injury (AKI) stage after 48 h in patients treated with dobutamine vs levosimendan. *Statistically significant difference. Figure drawn freely from the data reported by Guerrero-Orriach et al. [79]

The same research group [80] has also conducted a more conventionally configured RCT in 60 patients with confirmed LCOS after cardiac surgery, comparing the impact on kidney failure of levosimendan (0.1 μg/kg/min for 24 h) or dobutamine (starting infusion rate 5 μg/kg/min, thereafter variable according to response). Renal status was assessed using the acute kidney injury scale (AKIS). At LCOS diagnosis, NT-proBNP, stroke volume index (SVI), and central venous saturation were significantly (*p* < 0.005) lower in the dobutamine group, suggesting that cardiovascular status was worse in the levosimendan group. No significant differences in haemodynamic indices were noted at 48 h after the start of treatment but there were variations in AKIS at that time which favoured levosimendan. In total, 83% of the patients treated with dobutamine and 46% of patients in the levosimendan group exhibited no AKI at diagnosis, with a significant difference of 37% between groups. Twenty-four hours after treatment, the AKI stage improved in 23% of patients treated with levosimendan and 3% of patients treated with dobutamine, with a significant difference of 20% (*p* < 0.05). The authors concluded that the improvement in renal function is another effect of levosimendan that distinguishes it from beta-agonists (Fig. 5).

A further addition to the knowledge base in cardiac surgery is offered by the latest work of Ricksten, Lannemyr, and colleagues [81], who have examined the effect of levosimendan on renal blood flow (RBF) and GFR in patients with postoperative AKI. The 29 enrolled patients had preoperative serum creatinine levels within the normal range and postoperative AKI defined as an increase in serum creatinine of ≥ 27 μmol/l (or > 50% according to the Kidney Disease Improving Global Outcomes [KDIGO] criteria) in the 48 h after surgery. Patients were treated with placebo (*n* = 13) or levosimendan (*n* = 16; 12 μg/kg bolus then 0.1 μg/kg/min for 5 h) and derived renal function indices were determined at 3, 4, and 5 h. Relative to placebo, levosimendan increased RBF (due to a decrease in renal vascular resistance) but had no significant impact on GFR; filtration fraction decreased. These results suggest that, in AKI, levosimendan induces dilation of both pre- and post-glomerular resistance vessels. Intriguingly, this is in discordance with the previous results by the same group [82].

Last for attention in the context of cardiac surgery is a study by Zhang et al. [83] and analysis by Jawitz et al. [84] of data from the LEVO-CTS trial. The former determined, on the basis of retrospective data from 417 cardiac surgery patients treated at the Capital Medical University in Beijing, China, that perioperative administration of levosimendan was an independent protective factor for the development of AKI. By contrast, the LEVO-CTS researchers, drawing on findings from 854 patients, of whom 231 (27.0%) developed postoperative AKI, reported no association between levosimendan treatment and AKI (OR 0.92, 95% CnI 0.66–1.29).

In other developments, John and colleagues [85] have reported a comparison of the renal effects of levosimendan versus dobutamine in 58 adult patients with acute decompensated heart failure with reduced EF and impaired renal function. In this prospective, open-label trial, levosimendan was given as an initial 12 μg/kg bolus followed by infusion at 0.1 μg/kg/min for 24 h and dobutamine was started as a 5 μg/kg/min infusion (titratable to a maximum of 20 μg/kg/min). Cardiac and renal parameters were monitored at 48 h, 7 days, or discharge (whichever occurred first) and at day 30 post-treatment.

Levosimendan reduced ICU stay significantly (*p* < 0.038), whereas dobutamine decreased the duration of hospital stay (*p* < 0.015), a finding in alignment with that of Chen and co-workers [77, 78] above. There was a progressive decline in serum creatinine in the levosimendan group. In the dobutamine group, there was an initial decline followed by an increase, such that by day 30 there was a significant intergroup difference in favour of levosimendan (– 1.41 ± 1.93 vs + 0.76 ± 1.6 μmol/l; *p* < 0.001). In the levosimendan group, estimated GFR (eGFR) showed significant improvement from baseline to 48 h (*p* = 0.003) and to day 7 (*p* = 0.03) and the increment in eGFR versus dobutamine was statistically significant at day 30 (*p* = 0.001). There were three deaths among the 22 levosimendan-treated patients and 13 among the 36 randomized to dobutamine but no statistically significant intergroup differences at any point of testing.

A retrospective multi-institution database study has explored the effect of levosimendan in 1095 Taiwanese adult patients with AHF and reduced LVEF treated with levosimendan or dobutamine in cardiac critical care units between 2013 and December 2018 [86]. The 154 patients treated with levosimendan (0.05–0.2 μg/kg/min for 72 h; no loading dose) comprised 102 with eGFR ≥ 30 ml/min/1.73 m^2^ and 52 with eGFR < 30 ml/min/1.73 m^2^; corresponding numbers in the dobutamine cohort were 567 and 374. Patients requiring VA-ECMO were excluded. Raw data was adjusted by propensity scoring for variables including age, sex, LVEF, eGFR, use of inotropic agents (dopamine, norepinephrine, and epinephrine), MV during the index admission, and SOFA and Acute Physiology and Chronic Health Evaluation III scores. Mortality at 30, 90, and 180 days was examined, stratified by eGFR.

No significant differences in mortality rates were identified with the exception of propensity-matched in-hospital mortality in patients with eGFR ≥ 30 ml/min/1.73 m^2^, which was significantly lower in the levosimendan group (nine [11%] vs 25 [17.2%] deaths; OR 0.37; 95% CnI 0.15–0.92; *p* = 0.032). On the basis of all the data collected, it was concluded that, for patients meeting the study criteria, eGFR < 30 mL/min/1.73 m^2^ “is not necessarily a contraindication for levosimendan”.

A final recent contribution in this area comes from a study group that evaluated levosimendan for the protection of renal function in kidney transplant recipients [87]. Between April 2014 and December 2016, a total of 89 transplant recipients received either standard care or standard care plus levosimendan (6–12 μg/kg bolus, then 0.1 μg/kg/min for 1 h, then 0.2 μg/kg/min for 24 h). Since blinding arrangements were not reported and some paediatric patients may also have been enrolled, we do not comment on this paper.

### Experts’ assessment

The extant guidelines of the European Society of Intensive Care Medicine (ESICM) do not explicitly recommend the use of levosimendan for renal protection [88]. The authors characterize their advice as a recommendation with low-level evidence. The evidence in question derives principally from the LEVO-CTS and CHEETAH trials and from LEOPARDS. Meta-analyses considered during the development of the guidelines showed some renal-protective effects in both cardiac surgery and critical illness scenarios, but the effect was attenuated when calculations were restricted to higher quality studies such as the three aforementioned.

Broadly speaking, none of the newer meta-analyses fully addresses the issue of study quality and heterogeneity identified in the ESICM critique and none of the trials we have described is big enough or conclusive enough to outweigh the findings of the previous large trials in this area.

That said, several of the more recent studies have produced encouraging hints of possible renal-protective effects in both cardiac surgery and decompensated heart failure. This is important because worsening of renal function may be present in up to 40% of patients hospitalized for heart failure. We must appreciate that renal venous congestion is a major causal factor for a deterioration of kidney function [89] and here, the particular mode of action of levosimendan may offer benefits. Thus, the observed clinical findings are supported by a sound pathophysiological rationale.

While those findings need further investigation and validation in larger trials, it may be pertinent to note that, in the summary of their work, the authors of the ESICM guidelines [88] used the phrase “not using… levosimendan for kidney protection *solely*” [our emphasis added]. Surgery and cardiac decompensation are both areas where the use of levosimendan may be appropriate for reasons not directly or exclusively connected to renal protection but where any such ancillary effect could be considered a clinical bonus. Using levosimendan according to clinical judgement for its established haemodynamic and/or inotropic effects therefore seems to us to be a reasonable course of action. In this context, work such as that of Chan et al. [86] showing that low eGFR is not necessarily an obstacle to the use of levosimendan is instructive and reassuring, though we would advocate careful attention to existing Summary of Product Characteristics guidance on the use of this or any other drug in patients with confirmed renal dysfunction.

In short, further in-depth evaluation of the use of levosimendan requires additional trials in well-defined patient populations, and study designs should minimize the impact of patient management.

## Pulmonary hypertension and right ventricular dysfunction

### Recent meta-analyses

No meta-analyses were identified within our prespecified time range.

### Recent clinical trials

Recent additions to the dataset for levosimendan in PH include two reports from the Hemodynamic Evaluation of Levosimendan in Patients with PH-HF-pEF (HELP) trial (NCT 03541603) [90, 91]. The goal of HELP was to explore the efficacy and mechanism of action of i.v. levosimendan on haemodynamics in patients with PH and heart failure with preserved EF (HFpEF), a condition for which there are currently no proven treatments. The study had two parts. The first part was an open-label assessment of a 24-h infusion of levosimendan on resting and exercise haemodynamics with the patients serving as their own controls. The data revealed significant reductions in central venous pressure (CVP) and pulmonary wedge pressure (PCWP) at rest and exercise. Those patients who responded with a ≥ 4 mmHg decrease in PCWP with exercise were then enrolled in the second part of the study. To this end, patients (*n* = 37; 61% female; mean age 69±9 years) who displayed a satisfactory initial haemodynamic response to a 24-h open-label infusion of levosimendan (0.1 μg/kg/min) were randomized according to a double-blind schedule to a weekly home infusion of levosimendan (0.075–0.1 μg/kg/min/24 h) or placebo for 5 weeks. This part tested the durability of chronic levosimendan infusion and its effect on exercise capacity as determined by a 6-min walk test.

While there was not a significant treatment effect demonstrated for the primary efficacy outcome of decrease in PCWP during exercise, significant effects were seen on PCWP and CVP with a mixed-effects model using repeated measurements, along with a significant 29.3 m improvement in 6-min walk distance compared with placebo (*p* = 0.033) [90]. There was no change in CI at rest or with exercise between groups, nor was there any indication of increased elastance by echocardiography. An analysis of the data from a separate report of the HELP investigators showed that the reductions in CVP and PCWP at rest and exercise were explained by a reduction in stressed blood volume from vasodilation of the splanchnic circulation [91]. These data support the fact that levosimendan was effective as an adenosine triphosphate-sensitive potassium channel activator without inotropic effects.

Wang and colleagues [92] evaluated the effects of a 24-h infusion of levosimendan (i.v. bolus of 6–12 μg/kg, then 0.1 μg/kg/min infusion) in 59 patients with acute decompensated heart failure in a single-centre, randomized, controlled, open-label study conducted at Hebei Medical University, China, in 2017. Patients had NYHA class III or IV symptoms, LVEF < 45%, and tricuspid regurgitation detected by echocardiography. Levosimendan was compared with placebo on top of standard heart failure therapy as described in the previous version of the heart failure guidelines [93].

The use of levosimendan was associated with statistically significant (*p* ≤ 0.05) improvements (versus control and/or baseline) in a range of echocardiographic indices of RVF. LV end-diastolic diameter and LVEF were enhanced significantly. Among clinical indices, urinary output was significantly larger at the end of 24 h of levosimendan infusion than in the placebo group (inter-group mean difference ≈ 170 ml; *p* = 0.026) and mean BNP level was ≈ 350 ng/ml lower (*p* = 0.005). At 1-month follow-up, there was one recorded death in each group and one re-admission (in the placebo group). The average length of hospital stay was ≈ 2 days longer in the levosimendan group (*p* = 0.121).

Wang and colleagues cite, as a limitation of their study, the fact that “some of the patients were not given beta-blockers or other drugs because of heart rate, blood pressure and onset of acute heart failure” [92]. In fact, beta-blocker use was recorded in 90% of patients randomized to levosimendan, but in only 69% of those in the placebo group (*p* = 0.057). Rather than being a limitation, this imbalance may be seen as a reflection of the fact that levosimendan, unlike conventional adrenergic inotropes, remains effective in settings of beta-adrenergic blockade [94].

Based on data from a retrospective review of a single-centre transplant list, Tavares-Silva and colleagues [95] deemed levosimendan to be a safe and effective alternative to either iloprost or nitric oxide for PH reversibility assessment of heart transplant candidates but emphasized that their data offered no insights into whether the use of levosimendan in this way facilitated better selection of transplant candidates or was associated with any robust indication of improved outcomes from transplantation. Levosimendan was the only drug of the three reviewed that reduced pulmonary artery wedge pressure and CVP.

### Experts’ assessment

Hansen and colleagues [96] have previously reviewed experience with levosimendan in PH and their conclusion may be cited in extenso as a fair guide to current perspectives on this use of levosimendan: “The present literature on levosimendan in PH, despite limited in its extent, suggests that levosimendan is potentially favourable in treating PH and associated [right ventricular] failure resulting from different aetiologies such as [pulmonary artery hypertension, left heart disease and congenital heart disease]. The existing literature does not provide adequate evidence to currently recommend the use of levosimendan in PH and associated [right ventricular] failure”.

The studies and data on which this conclusion was based predate our self-imposed time cut-off. Subsequent additions to the literature are briefly summarized above. Those later studies are broadly encouraging along the lines identified by Hansen et al. but none is of sufficient scope or power to provide the basis of a formal recommendation. Further work in this area will therefore be needed to fully elucidate the possible applications of levosimendan. Insights from HELP are being used to develop a Phase III regulatory study in the USA and Europe.

In this context, Mauriat et al. [17] suggested that when levosimendan was used following surgery in GUCH patients with a high prevalence of RVF and PH, a beneficial impact on the durations of MV and ICU stay was noticed.

The authoritative review by Hoeper et al. [97] on the management of PH and RV failure notes the potential of levosimendan in this setting and its possible advantages over dobutamine in pre-clinical research but also notes the lack of comparative clinical data. Other authors highlight that in these settings inotropes that reduce cardiac filling pressures may be preferred (i.e. levosimendan or phosphodiesterase type III inhibitors) [98, 99].

Cholley and colleagues [100] argue, on haemodynamic grounds, that the use of levosimendan to treat RV failure should be considered with great care and we share their concerns. Some mitigation of that risk is inherent in the recommendation that patients with PH and right-sided heart failure requiring ICU therapy should be treated at expert centres capable of providing all treatment options (including lung transplantation).

## Discussion

As part of a broad-ranging review, Cholley et al. [100] assessed the evidence in favour of several of the indications we have examined. In the matter of prevention of organ dysfunction in sepsis, the verdict of those commentators was that there is no particularly strong evidence for benefit from levosimendan. For CS, weaning from VA-ECMO and TTS their verdict was more positive but still based on limited evidence. For postoperative LCOS those authors advised “Maybe” for isolated CABG but “No” for general cardiac surgery. Having examined additional data from the past 2 years, we think those assessments remain broadly a fair indication of the current position.

A current deficit of evidence is, of course, not permanent confirmation that an intervention is without effect. Fifty trials of levosimendan are currently registered as active at www.clinicaltrials.gov, many of which are concerned with the critical illness situations that we have reviewed. Important insights may emerge from these that may require a further re-evaluation of the use of levosimendan in one or more indications. We can note also that the weight of published data for levosimendan is already substantial to a degree that is unusual for i.v. cardio- and vasoactive drugs used in acute settings [101]. Indeed, and somewhat perversely, some established haemodynamically active i.v. interventions (e.g. dobutamine) are broadly used in ICU or EM settings with little or no robust experimental foundation. In this context, we draw attention to a survey by Carsetti and colleagues [102] of the use of vasopressors and inotropic drugs in critically ill patients, including those with septic shock and CS. Their data identifies a high level of agreement among ICU practitioners that the use of levosimendan “should be considered” on an individual patient basis.

Across the emergency and intensive care applications we have considered, significant numbers of patients may have to be enrolled in RCTs to demonstrate a robust effect on mortality. A response to the meta-analysis of Jaguszewski et al. [21] is instructive in this regard. This meta-analysis, based on data from 10 studies, identified a possible 30-day survival advantage of levosimendan over dobutamine in postoperative LCOS. However, Sanfilippo et al. [103] have calculated that the number of patients enrolled in this analysis (*n* = 2263) was 27% of the total required for a robust comparison.

Acquiring the larger numbers probably needed highlights both the endpoint(s) used to estimate those numbers and the question of heterogeneity of the patient population. We have alluded elsewhere in this paper to the question of whether mortality is the most appropriate outcome for evaluations of levosimendan in critical care. The candid short answer to that question is that we do not know whether mortality/survival is the most informative outcome, nor what time after event/treatment is the proper reflection of any true treatment effect. However, in the current state of knowledge, seeking to replace mortality with some other endpoint(s) may be to reformulate the question without bringing us closer to a solution. Moreover, the priority attached to mortality as a benchmark of effect by some regulatory agencies cannot easily be ignored. Unless or until alternative endpoints emerge from ongoing research, trials are likely to retain mortality as at least a secondary endpoint and their success is likely to be framed by any effect or lack of effect on this outcome [101].

Population heterogeneity and differences in management may be another consideration. The complex variable presentations of critical illness patients may aggregate substantially differing pathophysiologies and so mask a treatment effect in subgroups. Fully characterizing those who can benefit (and equally importantly those who will not) can be difficult; thereafter, recruiting enough optimally configured patients to a prospective trial may be a considerable logistic challenge. Additionally, it may be highly important to precisely characterize the haemodynamic pattern by invasive monitoring, i.e., right heart catheterization [104], and this was not the case in the majority of studies to date. Situations such as weaning from VA-ECMO or MV may be easier to navigate from this point of view but that remains to be proved.

For the moment, therefore, evidence for levosimendan in critical care applications is still underpowered. Rectifying that deficit will take time and ingenuity in trials design. Pending further research in these matters, we recommend that physicians adhere to expert guidelines and expert advice in deciding whether or not to use levosimendan as part of their medical response to a haemodynamic imbalance in a critical care situation. On the other hand, the evidence-set for levosimendan is currently among the largest available for any i.v. cardio- and vasoactive drugs used in ICU and EM [105]. The fact that other inotropes or inodilators have not been studied as much as levosimendan should weigh in on an “evidence-based” medicine environment.

## Conclusion

While waiting for more data, we give levosimendan a “should be considered” recommendation in ICU/EM settings with different levels of evidence in postoperative settings, septic shock, weaning from ventilator, weaning from VA-ECMO, CS, TTS, in cases when an inodilator is needed to restore acute severe reduced left- or right ventricular ejection fraction and overall haemodynamic balance, and also in the presence of renal dysfunction/failure. Since the development of levosimendan as therapy for PH associated with HFpEF is ongoing, we make no recommendation for that clinical setting.

## Data Availability

The data evaluated in this article are derived from freely available published sources, all of which are cited in the reference list.

## Abbreviations

AHF: Acutely decompensated chronic heart failure
AKI: Acute kidney injury
AKIS: Acute kidney injury scale
AMI: Acute myocardial infarction
BNP: Brain natriuretic peptide
CABG: Coronary artery bypass grafting
CHEETAH: Levosimendan in high-risk patients undergoing cardiac surgery (study acronym)
CnI: Confidence interval
CI: Cardiac index
CO: Cardiac output
CPB: Cardiopulmonary bypass
CS: Cardiogenic shock
CVP: Central venous pressure
eGFR: Estimated glomerular filtration rate
EM: Emergency medicine
ESICM: European Society of Intensive Care Medicine
GFR: Glomerular filtration rate
GLASSES: Levosimendan and Global Longitudinal Strain Assessment in Sepsis (study acronym)
GUCH: Grown-up congenital heart disease
HELP: Hemodynamic evaluation of levosimendan in patients with pulmonary hypertension and heart failure with preserved ejection fraction (study acronym)
HFpEF: Heart failure with preserved ejection fraction
HR: Hazard ratio
IABP: Intra-aortic balloon pump
ICS: Infarction-related cardiogenic shock
ICU: Intensive care unit
INTERMACS: Interagency registry for mechanically assisted circulatory support
IQR: Interquartile range
i.v.: Intravenous
KCCQ: Kansas City Cardiomyopathy Questionnaire
KDIGO: Kidney Disease Improving Global Outcomes
LCOS: Low cardiac output syndrome
LEOPARDS: Levosimendan to prevent acute organ dysfunction in sepsis (study acronym)
LEVO-CTS: Levosimendan in patients with left ventricular systolic dysfunction undergoing cardiac surgery on cardiopulmonary bypass (study acronym)
LH: Length of hospital stay
LICORN: Levosimendan in coronary artery bypass graft patients with poor left ventricular function (study acronym)
LV: Left ventricular
LVEF: Left ventricular ejection fraction
LVSWI: Left ventricular stroke-work index
MCS: Mechanical circulatory support
MV: Mechanical ventilation
NT: N-terminal
NT-proBNP: N-terminal pro-brain natriuretic peptide
NYHA: New York Heart Association
OR: Odds ratio
PCWP: Pulmonary capillary wedge pressure
PH: Pulmonary hypertension
RBF: Renal blood flow
RCT: Randomized controlled trial
REFSMD: Random-effects model standardized mean difference
RETAKO: Spanish National Registry on Takotsubo syndrome
RHBNP: Recombinant human brain natriuretic peptide
RR: Relative risk
RVF: Right ventricular failure
SBT: Spontaneous breathing test
SOFA: Sequential organ failure assessment
SSC: Surviving Sepsis Campaign
STEMI: S-T segment elevation myocardial infarction
SVI: Stroke-volume index
TTS: Takotsubo syndrome
VA-ECMO: Veno-arterial extracorporeal membrane oxygenation
VA-VA-ECMO: Double peripheral venous and arterial cannulation for extracorporeal membrane oxygenation
VIS: Vasoactive and inotropic score
WMD: Weighted mean difference
